# Evaluating otter reintroduction outcomes using genetic spatial capture–recapture modified for dendritic networks

**DOI:** 10.1002/ece3.8187

**Published:** 2021-10-07

**Authors:** Sean M. Murphy, Jennifer R. Adams, Lisette P. Waits, John J. Cox

**Affiliations:** ^1^ Wildlife Management Division New Mexico Department of Game & Fish Santa Fe New Mexico USA; ^2^ Department of Fish and Wildlife Sciences University of Idaho Moscow Idaho USA; ^3^ Department of Forestry and Natural Resources University of Kentucky Lexington Kentucky USA

**Keywords:** dendritic network, founder effect, *Lontra canadensis*, population density, recapture, river otter, spatially explicit capture

## Abstract

Monitoring the demographics and genetics of reintroduced populations is critical to evaluating reintroduction success, but species ecology and the landscapes that they inhabit often present challenges for accurate assessments. If suitable habitats are restricted to hierarchical dendritic networks, such as river systems, animal movements are typically constrained and may violate assumptions of methods commonly used to estimate demographic parameters. Using genetic detection data collected via fecal sampling at latrines, we demonstrate applicability of the spatial capture–recapture (SCR) network distance function for estimating the size and density of a recently reintroduced North American river otter (*Lontra canadensis*) population in the Upper Rio Grande River dendritic network in the southwestern United States, and we also evaluated the genetic outcomes of using a small founder group (*n* = 33 otters) for reintroduction. Estimated population density was 0.23–0.28 otter/km, or 1 otter/3.57–4.35 km, with weak evidence of density increasing with northerly latitude (β = 0.33). Estimated population size was 83–104 total otters in 359 km of riverine dendritic network, which corresponded to average annual exponential population growth of 1.12–1.15/year since reintroduction. Growth was ≥40% lower than most reintroduced river otter populations and strong evidence of a founder effect existed 8–10 years post‐reintroduction, including 13–21% genetic diversity loss, 84%–87% genetic effective population size decline, and rapid divergence from the source population (*F*_ST_ accumulation = 0.06/generation). Consequently, genetic restoration via translocation of additional otters from other populations may be necessary to mitigate deleterious genetic effects in this small, isolated population. Combined with non‐invasive genetic sampling, the SCR network distance approach is likely widely applicable to demogenetic assessments of both reintroduced and established populations of multiple mustelid species that inhabit aquatic dendritic networks, many of which are regionally or globally imperiled and may warrant reintroduction or augmentation efforts.

## Graphical abstract

Monitoring the demographics and genetics of reintroduced populations is critical to evaluating reintroduction success, but species ecology and the landscapes that they inhabit often present challenges for accurate assessments. Using genetic detection data collected via fecal sampling at latrines, we demonstrate applicability of the spatial capture–recapture (SCR) network distance function for estimating the size and density of a recently reintroduced North American river otter (*Lontra canadensis*) population in the Upper Rio Grande River dendritic network in the southwestern United States, and we also evaluated the genetic outcomes of using a small founder group for reintroduction. Combined with noninvasive genetic sampling, the SCR network distance approach is likely widely applicable to demogenetic assessments of both reintroduced and established populations of multiple mustelid species that inhabit aquatic dendritic networks, many of which are regionally or globally imperiled and may warrant reintroduction or augmentation efforts.

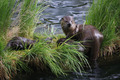

## 1 INTRODUCTION

Reintroduction has become an important tool for overcoming barriers to natural recolonization and reestablishing wildlife populations to historical ranges (Galetti et al., 2017; Seddon et al., 2007, 2014). Because of financial and logistical constraints, however, reintroductions are often conducted using small founder groups that have heightened vulnerability to demographic and environmental stochasticity (Brichieri‐Colombi & Moehrenschlager, 2016; Szűcs et al., 2017). Consequently, reintroduced populations sometimes exhibit deleterious demographic and genetic anomalies, including founder effects, that can increase the probability of extinction and reintroduction failure (Kanarek et al., 2015; Szűcs et al., 2017).

Monitoring the status of reintroduced populations via estimation of key demographic and genetic parameters, such as population size, density, and genetic diversity, is critical to evaluating reintroduction success and informing adaptive management strategies (DeMay et al., 2017; Ewen & Armstrong, 2007; Nichols & Armstrong, 2012; Robert et al., 2015; Seddon, 1999). For example, four of the five quantitative criteria used by the International Union for Conservation of Nature to assign extinction risk levels specifically require population size estimates (IUCN, 2012). Furthermore, general criteria for evaluating both short‐ and long‐term reintroduction success necessitate monitoring demographic and genetic characteristics of populations at predefined intervals (Robert et al., 2015; Seddon, 1999).

**FIGURE 1:**
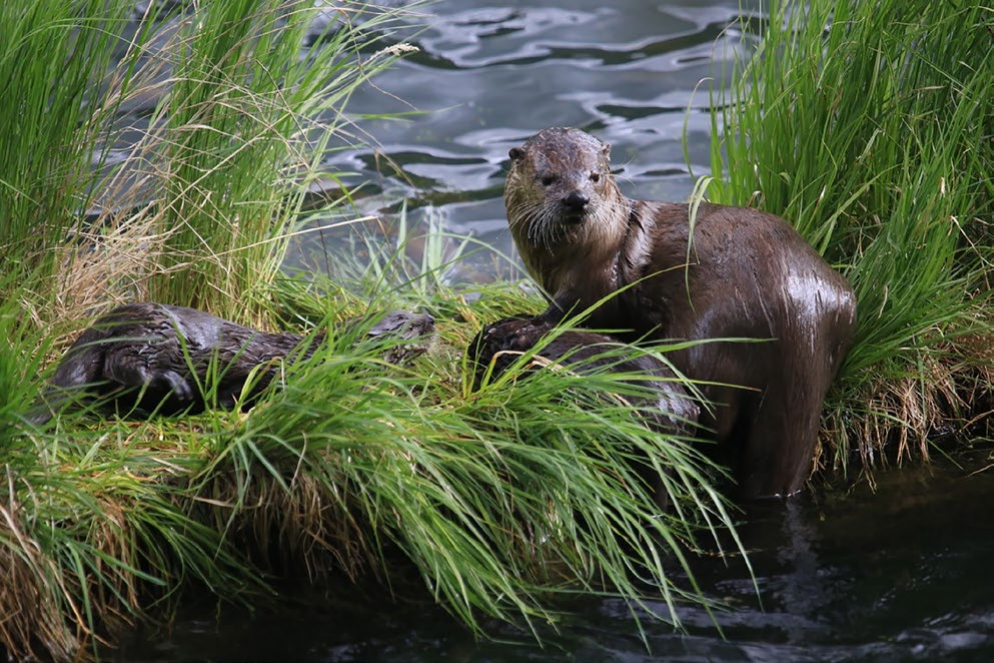
Two North American river otters (*Lontra canadensis*) in a riverine dendritic network. Photograph by John J. Cox, University of Kentucky

Yet, the intrinsic ecological characteristics of many wildlife species often present considerable difficulty in monitoring their populations. One such example is lutrinids, including North American river otters (*Lontra canadensis*; Figure 1), which are semiaquatic carnivores with strongly territorial but semi‐social behavior that display high site fidelity over surprisingly large areas relative to their body size (Hung & Law, 2016; Larivière & Walton, 1998; Rivera et al., 2019; Stevens & Serfass, 2008). Their expansive territories and movement patterns are primarily structured by the hydrographical systems that they inhabit, such as rivers and coastal shorelines, which represent hierarchical dendritic networks comprised of multiple branches of suitable habitats (Brown & Swan, 2010; Campbell Grant et al., 2007). This results in predominant movements by otters being approximately linear along the branches of a dendritic network, with tortuosity closely linked to the spatial orientation of branches (i.e., sinuous; Blundell et al., 2001; Quaglietta et al., 2014; Sauer et al., 1999).

The long‐range movement capabilities of otters necessitate sampling over large geographical areas, an often logistically challenging and financially prohibitive endeavor (Quaglietta et al., 2015), to prevent overestimation of population density that can result from truncation bias if the effective sampling area is too small relative to the true extent of animal movement (Fleming et al., 2021; Obbard et al., 2010; Tobler & Powell, 2013). Otters generally do not have individually unique natural markings, which prevents individual identification that is required for estimating demographic parameters from camera‐trapping, unless a portion of individuals are physically captured and given unique artificial marks (Murphy et al., 2019; Sollmann et al., 2013; Whittington et al., 2018). Non‐invasive genetic sampling of scats deposited at latrines is an efficient method for surveying otter populations, but otter diet and their tendency to defecate on exposed features result in fecal samples having notoriously high DNA degradation rates, poor amplification rates, and non‐negligible genotyping error (Aristizábal Duque et al., 2018; Klütsch & Thomas, 2018; Lerone et al., 2014). The presumably high site fidelity of otters to multiple latrines (Gorman et al., 2006; Rivera et al., 2019; Stevens & Serfass, 2008) results in researchers approximately sampling with replacement when using non‐invasive scat sampling, because an individual otter can visit multiple latrines multiple times within a single sampling occasion; however, these highly informative multi‐site detection data are discarded when using traditional non‐spatial models to estimate demographic parameters (e.g., Brzeski et al., 2013; Godwin et al., 2015). Furthermore, for species whose movements are predominantly constrained to within structured dendritic networks, not accounting for such restricted space use can severely bias estimates of population size, density, and thus population growth rate (Efford, 2019; Royle et al., 2013; Sutherland et al., 2015), potentially leading to flawed conservation and management.

The advent and refinement of spatial capture–recapture (SCR) models have addressed many of the sampling and analytical challenges presented by the unique spatial ecology of multiple wildlife species. Whereas traditional non‐spatial capture–recapture models use individual‐by‐occasion detection data to estimate population size, which necessitates ad hoc delineation of an often ill‐defined effective sampling area to derive population density, SCR models use individual‐by‐trap‐by‐occasion detection data to model the probability of detection at a given detector as a function of spatial proximity with an individual's activity center, and SCR models explicitly define the geographical area to which estimated density applies (Borchers & Efford, 2008; Efford, 2004; Efford & Fewster, 2013; Royle et al., 2014). This improved approach with SCR links population density with animal space use, which also allows much more flexibility in study designs compared with traditional non‐spatial models, such that irregularly spaced arrays of detectors can be used to efficiently sample populations and reliably estimate their densities across large spatial extents (Clark, 2019; Humm et al., 2017; Murphy et al., 2019).

Nevertheless, despite numerous advancements over nearly two decades, no published studies have applied SCR models to estimate population size or density of any otter species (but see Forman [2015]). This is perhaps because the predominately linear or sinuous nature of otter space use in branched dendritic networks strongly conflicts with how movement is modeled in a typical SCR model, namely, a two‐dimensional Euclidean distance model that assumes home ranges are approximately circular, irrespective of habitat or landscape structure (Efford, 2019; Royle et al., 2013). However, relatively recent SCR model extensions and alternative specifications now allow reliable population size and density estimation for species with non‐circular home ranges or structured space use. For example, the ecological distance model relaxes the Euclidean assumption by modeling animal movement as a function of least‐cost paths (Royle et al., 2013; Sutherland et al., 2015), and the anisotropic detection function transforms the detection functions for all animals to the predominant directionality of habitat or landscape structure, such that the aligned home ranges become circular (Efford, 2019; Murphy et al., 2016). More recently, the network distance function was developed, which employs a non‐Euclidean distance model that reflects the actual linear or sinuous distances along landscape features (Efford, 2017, 2019). When combined with appropriate ecological restriction of the state space, the network distance function can be used to estimate population size and density that better reflect the structural reality of dendritic networks and animal space use within them (Leuenberger et al., 2019; Warbington & Boyce, 2020).

Herein, we applied the SCR network distance approach to genetic detection data collected non‐invasively from a recently reintroduced North American river otter population that inhabited a severely cliff‐bounded dendritic network. We estimated population size, density, sex ratio, and derived a population growth rate estimate since the founder event 8–10 years prior. We also applied population genetic analyses to evaluate changes in genetic diversity and effective population size since the reintroduction occurred that used a small founder group. We demonstrate the effectiveness of non‐invasive genetic sampling and the SCR network distance approach for concomitantly evaluating demographics and genetics of populations that occupy highly structured dendritic networks, which should be applicable to multiple taxa.

## 2 MATERIALS AND METHODS

### 2.1 Reintroduction and study area

River otters were extirpated from most of the southwestern United States by the 1950s. Between 2008 and 2010, 33 river otters (unknown age or sex ratio) were translocated from the Puget Sound, Washington, to the Upper Rio Grande River Basin (URG) in northern New Mexico (Savage & Klingel, 2015). The URG was devoid of conspecifics at the time of reintroduction, and the nearest known otter population was ~250 km away in a disjunct watershed. Although founders were not radio‐monitored, >170 otter sightings and reproduction were documented post‐reintroduction, suggesting that population growth has occurred (Colorado Parks & Wildlife, 2018; Converse et al., 2014; Long, 2010; Savage & Klingel, 2015).

**FIGURE 2:**
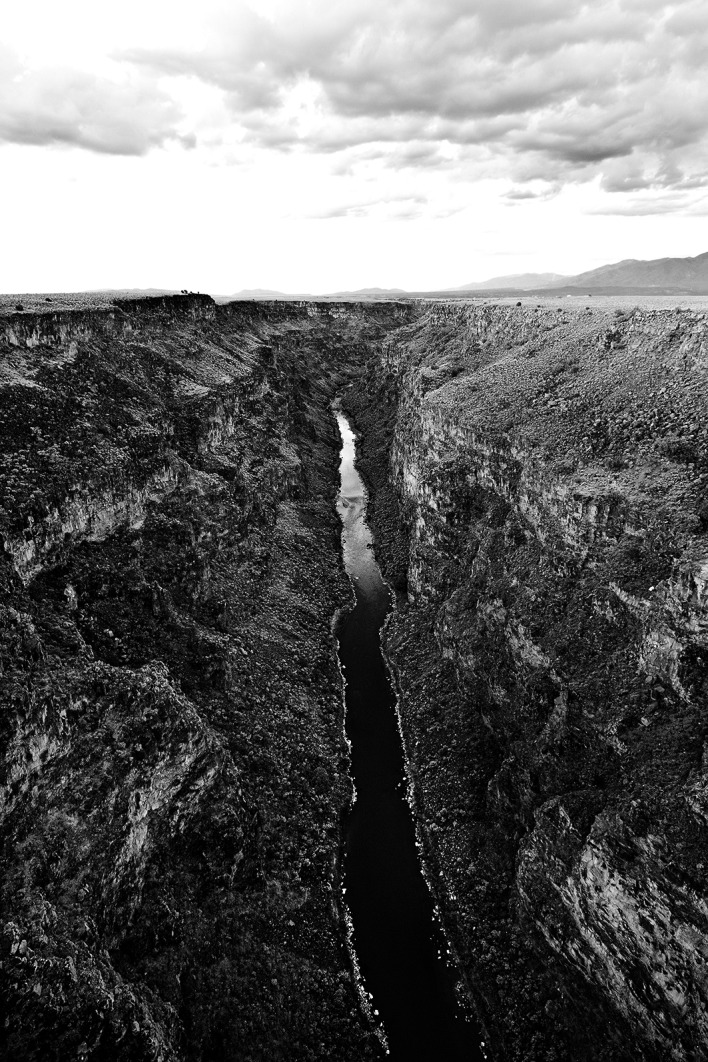
The Rio Grande River flowing through the Rio Grande Gorge in northern New Mexico, USA, depicting the landscape structure of the riverine dendritic network where river otter sampling occurred relative to the surrounding Taos Plateau. Photograph by Robert Wojtowicz; used with licensed permission

The URG dendritic network is located in the north–south trending Rio Grande Rift, which functions as the geologic boundary between the Colorado Plateau and interior North America. A dominant feature is the Rio Grande Gorge, an extensive canyon with cliffs up to ~240‐m high and within which flows the Rio Grande River, an IUCN Category V waterway (Figure 2). Predominant woody vegetation cover along perennial waterways includes cottonwood *Populus deltoides*, desert willow *Chilopsis linearis*, and salt cedar *Tamarix* spp., whereas semi‐arid shrublands and grasslands dominate the surrounding Taos Plateau (Griffith et al., 2006; Ruhlman et al., 2012). Elevations in our study area range from 1,831 to 2,261 m. The area is considered semi‐arid, receiving an average of only 15–25 cm of annual precipitation, with temperatures varying substantially by season and elevation, ranging from a low of −26℃ during winter to a high of 32℃ during summer.

### 2.2 Genetic sampling

**FIGURE 3:**
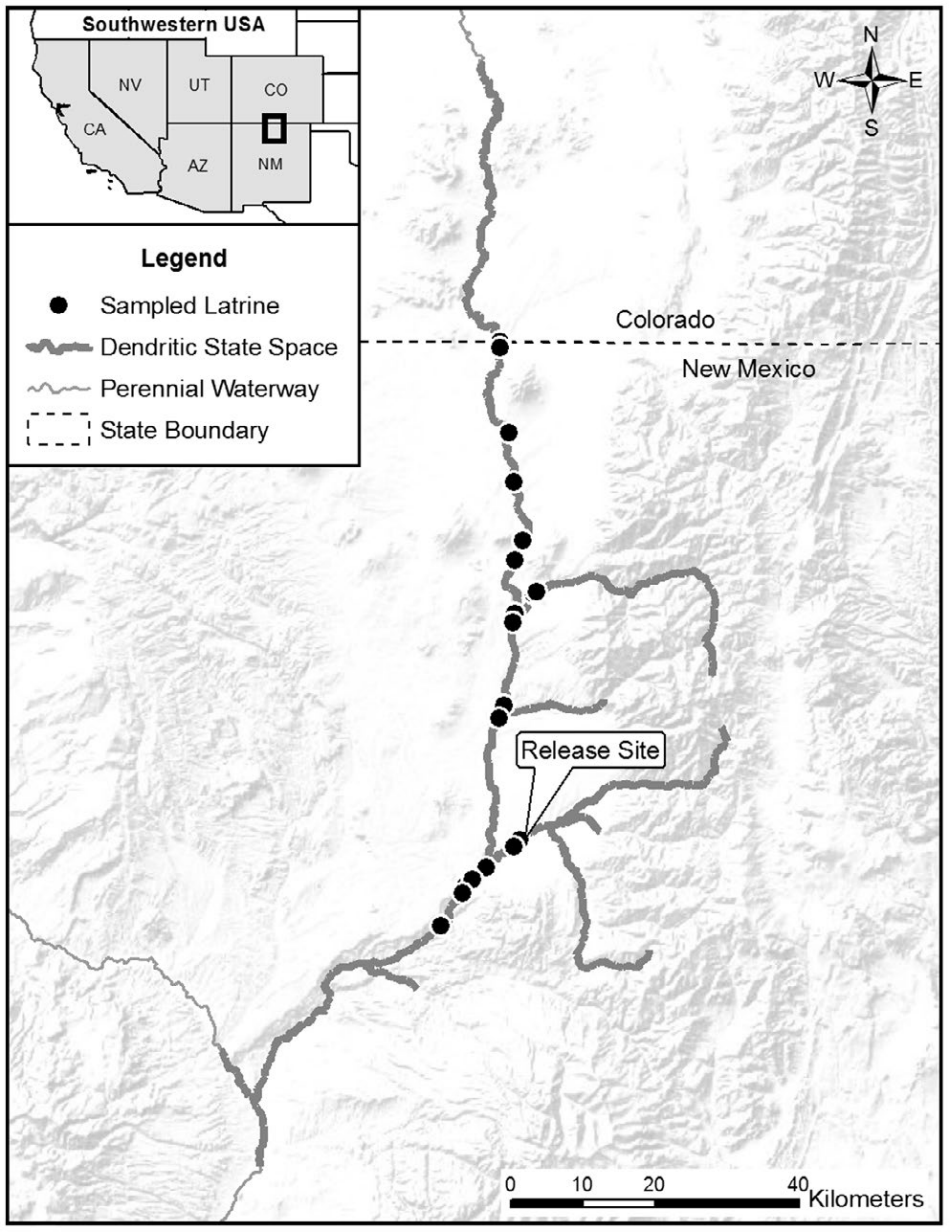
Locations of river otter latrines where fecal samples were collected from scats in the Upper Rio Grande dendritic network of perennial rivers and streams during 2018. A limited reintroduction occurred during 2008–2010 in which 33 founder otters were released at a single site. To estimate population size, density, and sex ratio since the founder event, a dendritic state space comprised of 359 river km was used with a network distance function in spatial capture–recapture models

During 2018, we conducted non‐invasive fecal DNA sampling of otter scats at active latrines located along 259 km of the URG dendritic network using a capture–recapture study design (Figure 3; Brzeski et al., 2013; Mowry et al., 2011). To maximize detection rates, we conducted sampling during February–April, which was within the typical river otter breeding season when latrine visitation and defecation rates are highest (Mowry et al., 2011; Rivera et al., 2019). This period is also characterized by cold, dry weather that generally corresponds to higher genotyping success of otter fecal DNA samples (Aristizábal Duque et al., 2018; Arrendal et al., 2007).

An initial 14‐day scouting period was conducted to find active latrines via a combination of foot and watercraft patrols. All scats were cleared from located latrines during this scouting period to equalize baseline detection rates across survey occasions (Morin et al., 2016; Murphy et al., 2018). Immediately thereafter, we initiated the capture–recapture survey during which latrines were revisited at seven‐ to ten‐day intervals for eight consecutive survey occasions. We collected two fecal samples from each scat and anal jelly (anal sac secretions) by extracting a ~0.5‐cm^3^ portion of the outside of scat using tweezers and ~0.5 ml of jelly using a metal spoon and placed samples in individually labeled vials containing 1.4 ml of DETS buffer (Morin et al., 2016; Murphy et al., 2018; Stenglein et al., 2010). To prevent cross‐contamination, we sterilized tweezers and spoons between sample collections using a lighter flame. After collecting fecal samples, we removed all scat and anal jellies from each latrine to prevent double sampling in subsequent occasions.

We also acquired tissue samples from river otters in the Washington source population (WA). Those samples were collected by Washington Department of Fish and Wildlife biologists from otters that were legally harvested during the 2017–2019 seasons. We used these tissue samples only in genetic analyses to investigate potential differences between the source and reintroduced populations.

### 2.3 Microsatellite genotyping

All collected samples were processed at the Laboratory for Ecological, Evolutionary and Conservation Genetics at University of Idaho (Moscow, USA) for DNA extraction, PCR amplification, and microsatellite genotyping. Genotyping all collected fecal samples was financially prohibitive, so we used the following randomized subsampling protocol: (1) divided the sample set in half based on the two samples that were collected from each scat, to prevent duplication; (2) randomized samples collected at each latrine within a given occasion; (3) randomly selected 3–4 samples from each latrine within each occasion for genotyping; and (4) if the first selected sample failed genotyping, then we attempted genotyping of the corresponding duplicate sample. Simulations by Murphy et al. (2016) demonstrated that such randomized subsampling has a nominal influence on SCR parameter estimates, primarily because the order of detection is not as critical in SCR compared with non‐spatial capture–recapture models (Augustine et al., 2014).

DNA was extracted from tissue and fecal samples using DNeasy Blood and Tissue Kits and QIAmp Fast DNA Stool Mini Kits (Qiagen, Inc.), respectively. Fecal samples were extracted in a separate laboratory dedicated to low quality, low quantity DNA sources, and one negative control was included in each extraction to monitor reagent contamination. Twelve candidate otter‐specific microsatellite loci were evaluated (Beheler et al., 2004, 2005; Dallas & Piertney, 1998; Mowry et al., 2011); however, locus RIO03 was monomorphic in the tissue samples and locus RIO11 failed to amplify. Therefore, we used a 10‐locus multiplex to obtain genotypes: RIO01, RIO02, RIO04, RIO06, RIO07, RIO08, RIO12, RIO13, RIO16, and Lut453, as well as the SRY2 sex marker (Dallas et al., 2000). Four to six replicate PCRs were performed for the fecal samples that consistently amplified after an initial screening step of two amplifications; the tissue samples were amplified in duplicate. PCR products were visualized using a 3130xl DNA Sequencer and allele sizes were scored using Genemapper 5.0 (Applied Biosystems). Sample quality assessment and genotype screening methods followed those described by Stenglein et al. (2010). In short, we developed consensus genotypes for each sample by requiring an allele be detected in two independent PCRs to confirm a heterozygote and an allele be detected in three independent PCRs to confirm a homozygote. The SRY2 sex marker amplifies a fragment in males but not females (Dallas et al., 2000); we required 3–6 replicates for sex determination using this marker. If ≥2 replicates detected the Y chromosome, the sample was classified as a confirmed male, whereas if one replicate amplified the Y chromosome, we classified the sample as unconfirmed male because the Y chromosome amplification result was not confirmed. If no replicates amplified the Y chromosome, we classified the sample as female because 8–10 loci worked, thereby indicating sufficient DNA in the sample to avoid allelic dropout of the Y chromosome across 3–6 replicates.

Probability of identity for siblings (PI_sibs_) was calculated separately for tissue and scat genotypes using GenAlEx v6.5 (Peakall & Smouse, 2012). PI_sibs_ ≤ 0.01 was used as a cutoff for the number of loci required to distinguish among unique genotypes; to determine the number of unique genotypes, matching analysis was conducted using GenAlEx (Stenglein et al., 2010). Two fecal samples were conservatively considered as originating from the same individual if their locus‐specific alleles matched across ≥8 loci, and also if two fecal samples matched at only eight loci but the mismatches at locus nine or 10 were likely due to allelic dropout. If the 8‐ to 10‐locus consensus genotype matched another sample, but the sex results differed, we conservatively retained the samples as a match and the sex of the individual as male. We calculated genotyping error rates from the first two PCR replicates of fecal samples that had consensus genotypes at 8–10 loci, following the methods of Broquet and Petit (2004).

### 2.4 Latrine site fidelity

To characterize individual‐level latrine site fidelity, or the tendency of an otter to return to a latrine at which it was previously detected, we calculated a standardized site fidelity index (SSFI) that was developed specifically for estimating site fidelity from capture–recapture data (Tschopp et al., 2018). This approach represents a composite index that incorporates information on an animal's occurrence (IO; proportion of recaptures), permanence (IT; time spent at a site), and periodicity (It; recurrence at a site) relative to the duration of a capture–recapture survey (Haughey et al., 2020; Tschopp et al., 2018). Although the SSFI was developed to estimate population‐level site fidelity, we were interested in individual‐level site fidelity; therefore, we modified the formula for estimating SSFI with two alterations: (1) by changing the calculation of IO from the total proportion of recaptures of an individual during the survey to the total proportion of recaptures of a given individual at a given latrine during the survey; and (2) by changing the calculation of IT from the time between the first capture and last capture of an individual during the survey to the time between the first capture and last capture of a given individual at a given latrine during the survey. An individual's SSFI was then calculated using the IH4 method described by Tschopp et al. (2018), which is based on the harmonic mean:

$$\text{SSFI =}\;\frac{2}{\frac{1}{\text{IT}}+\frac{1}{\text{It}}}.$$

This SSFI is scaled 0–1, representing a continuum from low to high site fidelity. We subdivided individuals by sex to produce sex‐specific mean SSFIs. Additionally, we fit a generalized linear model with Poisson error distribution to the total number of latrines that each identified individual was detected to evaluate differential latrine use between sexes; inference was made based on estimated incident rate ratios (IRR; Cameron & Trivedi, 1998).

### 2.5 Population genetics analyses

We used the Genepop package (Rousset, 2008) in the R statistical computing environment (R Core Team, 2020) to test for Hardy–Weinberg equilibrium and quantify linkage disequilibrium; we ran 1,000 Markov chain iterations for each of 100 batches. We used the diveRsity package (Keenan et al., 2013) in R to estimate allelic richness via rarefaction, observed and expected heterozygosity, and inbreeding coefficients; we calculated 95% confidence intervals using 1,000 bootstrap iterations. We followed Waples et al. (2014) to estimate the genetic effective number of breeders and genetic effective population sizes via the linkage disequilibrium method for iteroparous species, starting with effective number of breeder estimates from N_E_ESTIMATOR v2.01 (Do et al., 2014). Two vital rates are used in this approach to correct for iteroparity, age at maturity and adult life span, which collectively explain most variation in genetic effective sizes (Waples, 2016; Waples et al., 2013, 2014). Data for those vital rates were unavailable from otters in the reintroduced or source populations, so we used averaged estimates among river otter populations throughout North America as surrogates (Larivière & Walton, 1998).

We tested for a genetic bottleneck using BOTTLENECK v1.2.02 (Piry et al., 1999), evaluating departure from mutation‐drift equilibrium via a two‐phase model that incorporated 30% of multi‐step mutations to account for uncertainty in the microsatellite mutation process (Luikart & Cornuet, 1998; Luikart et al., 1998; Peery et al., 2012). We ran 10,000 replicates and assessed support using Wilcoxon sign‐rank tests (Peery et al., 2012). We estimated pairwise genetic differentiation (*F*_ST_; Weir & Cockerham, 1984) between the source and reintroduced populations using the diveRsity package. Biologically important *F*_ST_ estimates were conservatively considered to have confidence interval lower bounds >0 and point estimates ≥0.05 (Hartl & Clark, 1997), considering the findings of other river otter genetic studies (Mowry et al., 2015).

### 2.6 Spatial capture–recapture analysis

Using only fecal genotypes from the URG population, we estimated population size and density with SCR models implemented by maximum likelihood in the R package secr (Borchers & Efford, 2008; Efford, 2004, 2021; Efford & Fewster, 2013). We modeled latrines as ‘count’ detectors for which the detection process followed a Poisson distribution, because latrines were spatially fixed and multiple otters could have visited the same latrine or multiple latrines multiple times during an occasion (Royle et al., 2014). We fit models with a hazard half‐normal detection function that had two primary parameters, the baseline detection rate at an otter's activity center (*λ*_0_) and the spatial scale of detection (*σ*). We accounted for varying survey effort among latrines and occasions (due to weather and logistics) by employing hazard‐based adjustments (Efford et al., 2013). We used the R package secrlinear (Efford, 2017) to replace the Euclidean distance model with the non‐Euclidean network distance function that represented the actual sinuous distances of the URG dendritic network, derived from digital spatial data of perennial waterways (U.S. Geological Survey, 2019). We specified a one‐dimensional state space with 100‐m point‐spacing resolution that was restricted to the dendritic network, extending approximately 4× *σ* beyond surveyed latrines (Efford, 2017; Royle et al., 2014).

We fit a priori models with and without the following potential sources of non‐spatial heterogeneity on detection function parameters: (1) a latrine‐specific behavioral response, bk, because of high latrine fidelity often exhibited by otters; and (2) sex, because male and female otters may have differential detection rates as a result of males often being the wider‐ranging sex (Larivière & Walton, 1998). We modeled sex as two‐class finite mixtures (Pledger, 2000), additive and interactive effects between bk and sex on *λ*_0_, and only sex on *σ*. In addition to fitting models in which otter density followed a homogeneous Poisson point process, we also fit inhomogeneous Poisson point process models in which otter density spatially varied along the dendritic network as a log‐linear function of latitude or distance (meters) from the reintroduction release site (Murphy et al., 2016).

We used Akaike's information criterion corrected for small sample size (AIC*_c_*) for model selection and considered all models ≤2 ΔAIC*_c_* of the top‐ranked model competing (Burnham & Anderson, 2002). If multiple models met this threshold and no uninformative parameters were evident, then we model‐averaged competing models to produce parameter estimates (Arnold, 2010). Additionally, we conducted goodness‐of‐fit testing of the top‐ranked model by first simulating 100 replicates of detection data for a hypothetical population with our resulting parameter estimates via the sim.linearpopn() and sim.capthist() functions in the R packages secrlinear and secr, respectively (Efford, 2017, 2021). We then refit the top‐ranked model to each replicate, obtained the average model deviance divided by the residual degrees of freedom (*W*), and estimated the probability of observing a value that large given the null hypothesis that data were generated under the model (see the secr.fit() function in the R package secr [Efford, 2021]).

To estimate the average annual population growth rate since the founder event, we used the exponential growth model described by Gotelli (2008). This assumes that density‐dependent population regulation is absent and that carrying capacity had not yet been reached when our survey occurred (Murphy et al., 2015, 2016). We optimistically assumed that all founding otters survived post‐translocation and specified 33 otters as the initial population size.

## 3 RESULTS

### 3.1 Genetic sampling

We located and surveyed 20 individual latrines along 259 km of perennial waterways in the URG; average spacing between latrines along the dendritic network was 5.52 km. We collected 1184 fecal samples from 622 individual scats and anal jellies ($\bar{x}$ = 1.90 samples/scat). An average of 31 scats or jellies were sampled at each latrine across the entire survey (range: 0–45 scats/latrine/occasion). We received tissue samples from 19 individual otters in the WA source population that were collected during 2017–2019.

### 3.2 Microsatellite genotyping

After accounting for duplicate samples, our randomized subsampling protocol resulted in the selection of 543 fecal samples for genotyping. Although this represented 46% of all samples collected, the effective subsampling corresponded to the selection of 87% of sampled scats for genotyping. For WA tissue samples, seven loci were the minimum necessary to distinguish among unique genotypes (PI_sibs_ = 0.002–0.005; Appendix S1: Table A1). However, several URG fecal sample genotypes differed at only one locus, suggesting that PI_sibs_ differed between the WA and URG populations. Thus, we recalculated PI_sibs_ for the fecal samples, which resulted in ≥8 loci being necessary to distinguish among unique genotypes (PI_sibs[8 loci]_ = 0.005–0.013; PI_sibs[9 loci]_ = 0.003–0.005; Appendix S1: Table A2).

All 19 tissue samples from the WA source population were successfully genotyped at 10 loci. Consensus genotypes at 8–10 loci were obtained for 77 total fecal samples from the reintroduced URG population, representing a 14% genotyping success rate. Six of those 77 fecal samples (7.80%) had consensus data at eight loci, two of which did not meet *PI*_sibs_ ≤0.01. Some groups of genotypes differed at only one locus, indicating that eight loci may have been insufficiently conservative. Therefore, we present results for both conservative and lenient matching rules; the matching rules for lenient genotypes entailed splitting genotypes that differed at only one locus into separate individuals. These rules resulted in a conservative detection history of 30 otters (12 M, 16 F, 2 unconfirmed M) that were detected 77 total times and a lenient detection history of 37 otters (17 M, 18 F, 2 unconfirmed M) that were detected 77 total times. A total of 41 detections (53%) in the conservative dataset were recaptures ($\bar{x}$ = 1.37 recaptures/individual; range: 0–8) and 30 of those were spatial recaptures ($\bar{x}$ = 1 spatial recapture/individual; range: 0–6), whereas a total of 32 detections (42%) in the lenient dataset were recaptures ($\bar{x}$ = 0.86 recapture/individual; range: 0–3) and 26 of those were spatial recaptures ($\bar{x}$ = 0.70 spatial recapture/individual; range: 0–2).

Estimated false allele and allelic dropout rates for the fecal samples were 5% and 29%, respectively (Appendix S1: Table A3). The uncertainty surrounding sex identification for two unconfirmed males was the direct consequence of the SRY method that is commonly used for otters (Dallas et al., 2000), which attempts to amplify the male Y chromosome and results in a positive male amplification and no PCR products for females. The method suffers from the fact that a negative amplification could be either a male with allelic dropout or a female (Mowry et al., 2011; Statham et al., 2007). As noted in the Methods, we conservatively labeled an individual as male if any sample in group samples for an individual was identified as a confirmed male based on ≥2 amplifications of the SRY locus.

### 3.3 Latrine site fidelity

Based on the conservative detection history, mean SSFI ranged from 0.10 for females (95% CI: 0.01–0.20) to 0.14 for males (95% CI: 0.01–0.28). Based on the lenient detection history, mean SSFI ranged from 0.06 for females (95% CI: 0.00–0.13) to 0.05 for males (95% CI: 0.00–0.11). Poisson regression models estimated that, on average, male otters were detected at ~2× more latrines per individual than female otters (IRR_Cons_ = 2.05 [95% CI: 1.20–3.60], *p* = .01; IRR_Len_ = 1.70 [95% CI: 1.02–2.92], *p* = .04), thereby suggesting differential latrine use between sexes.

### 3.4 Population genetics analyses

We detected violation of Hardy–Weinberg equilibrium at loci RIO12 and RIO13 in the lenient genotypes, following Bonferroni correction (*α* < .005), and linkage disequilibrium in 4% and 8% of pairwise loci comparisons in the conservative and lenient genotypes, respectively, following Bonferroni correction (*α* < .001). Genetic diversity estimates indicated a 17% decline in allelic richness and a 13%–21% decline in observed and expected heterozygosity in the reintroduced population (Table 1). All inbreeding coefficient estimates were negative, suggesting otters were less related than expected under a random mating model. Following adjustments for iteroparity, 84%–87% declines in effective number of breeders and effective population size in the reintroduced population were strongly supported. Statistical support existed for a genetic bottleneck in the reintroduced population, based on both the conservative (*p* = .006) and lenient genotypes (*p* = .01). A moderate, biologically significant level of genetic differentiation existed between the source and reintroduced populations, based on both conservative and lenient genotypes (*F*_ST(cons)_ = 0.09 [95% CI: 0.05–0.13]; *F*_ST(len)_ = 0.10 [95% CI: 0.06–0.14]).

**TABLE 1:** Measures of population genetic diversity, genetic fitness, and non‐random mating for river otters in the source (WA) and reintroduced (URG) populations (2017–2019)

Parameter^a^	Population		
	WA source (*n* = 19)	URG conservative (*n* = 30)	URG lenient (*n* = 37)
*A_R_*	4.44 (3.85–5.03)	3.70 (2.84–4.56)	3.69 (2.87–4.51)
*H_O_*	0.69 (0.63–0.75)	0.60 (0.48–0.72)	0.57 (0.46–0.68)
*H_E_*	0.68 (0.63–0.73)	0.54 (0.46–0.62)	0.53 (0.44–0.61)
*N_B_*	95 (31–∞)	15 (10–23)	12 (7–18)
*N_E_*	59 (19–∞)	9 (6–14)	8 (4–11)
*F*_IS_	−0.01 (−0.09 to 0.06)	−0.10 (−0.23 to 0.03)	−0.07 (−0.19 to 0.04)

Note

Two estimates are provided for the reintroduced population based on genotypes from conservative and lenient matching rules. Sample sizes of unique genotypes (*n*) are provided in parentheses next to each population data set and 95% confidence intervals are in parentheses next to each point estimate; infinity is denoted by ∞.

^a^ Allelic richness (*A_R_*), observed heterozygosity (*H_O_*), expected heterozygosity (*H_E_*), effective number of breeders (*N_B_*), effective population size (*N_E_*), and inbreeding coefficient (*F*_IS_).

### 3.5 Spatial capture–recapture analysis

We fit the same set of models to both conservative and lenient detection histories. Models that allowed otter density to spatially vary as a function of distance from the release site failed to converge (variance–covariance matrices contained zeros) and were therefore excluded from our final set of candidate models. Four candidate models were ≤2 ΔAIC*_c_* for both detection histories, with density as a homogeneous Poisson point process, *λ*_0_ varying by sex, and *σ* shared between sexes as commonalities (Table 2; Appendix S2: Tables B1 and B2). The top, most parsimonious model was identical for both detection histories, and the second‐ranked model differed only by whether an additive or interaction effect was specified between a latrine‐specific behavioral response (bk) and sex. One competing model for the conservative detection history included a density–latitude relationship that suggested otter density increased northward (*β*_Lat_ = 0.33), but the confidence interval reflected uncertainty about this effect (95% CI: −0.18 to 0.83). A latrine‐specific behavioral response that varied by sex was strongly supported; two competing models for the conservative detection history included these as additive or interaction effects, and one competing model for the lenient detection history included these as an interaction. Sex‐specific *σ* was present in only one competing model for the lenient detection history and none of the competing models for the conservative detection history, thereby strongly supporting movements by male and female otters were similar. Goodness‐of‐fit tests suggested that the top‐ranked models well fit both the conservative (*W*_Cons_ = 18.25, *W*_Sim_ = 11.45, *p* = .29) and lenient (*W*_Len_ = 14.49, *W*_Sim_ = 8.98, *p* = .23) detection data.

**TABLE 2:** Spatial capture–recapture model selection from analysis of conservative and lenient detection histories for the reintroduced river otter population in the Upper Rio Grande dendritic network (2018)

Model	*K* ^a^	AIC^b^	AIC*_c_* ^c^	ΔAIC*_c_* ^d^	logLik^e^	Deviance^f^	*w* ^g^
Conservative							
*D*~1 *λ*_0_~Sex *σ*~1	5	527.03	529.53	0.00	–258.17	516.34	0.31
*D*~1 *λ*_0_~bk + Sex *σ*~1	6	526.92	530.56	1.04	–257.14	514.28	0.18
*D*~Lat *λ*_0_~Sex *σ*~1	6	527.48	531.13	1.60	–257.48	514.96	0.14
*D*~1 *λ*_0_~bk × Sex *σ*~1	7	527.57	531.23	1.69	–257.79	515.58	0.10
Lenient							
*D*~1 *λ*_0_~Sex *σ*~1	5	545.16	549.02	0.00	–265.58	531.16	0.19
*D*~1 *λ*_0_~bk × Sex *σ*~1	7	547.56	549.49	0.47	–268.78	537.56	0.15
*D*~1 *λ*_0_~1 *σ*~Sex	5	548.18	550.11	1.09	–269.09	538.18	0.11
*D*~1 *λ*_0_~1 *σ*~1	4	549.47	550.72	1.70	–270.73	541.46	0.08

Note

Primary model parameters were population density (*D*), baseline detection rate (*λ*_0_), and the spatial scale of detection (*σ*). Models were fit in which *D* followed a homogenous Poisson point process (1) or spatially varied as a log‐linear function of latitude (Lat). Models also considered a latrine‐specific behavioral response (bk) that was either shared between sexes (1) or was sex‐specific (Sex), via both additive (+) and interaction (×) effects, and considered *σ* that was either sex‐specific (Sex) or shared between sexes (1). For brevity, only competing models (≤2 ΔAIC_c_) are presented; Tables B1 and B2 in Appendix S2 provide the complete model selection for both detection histories.

^a^ Number of model parameters.

^b^ Akaike's information criterion.

^c^ AIC corrected for small sample size.

^d^ Difference between AIC_c_ of model and AIC_c_ of top‐ranked model.

^e^ Log‐likelihood.

^f^ −2 × log‐likelihood.

^g^ Model weight.

**FIGURE 4:**
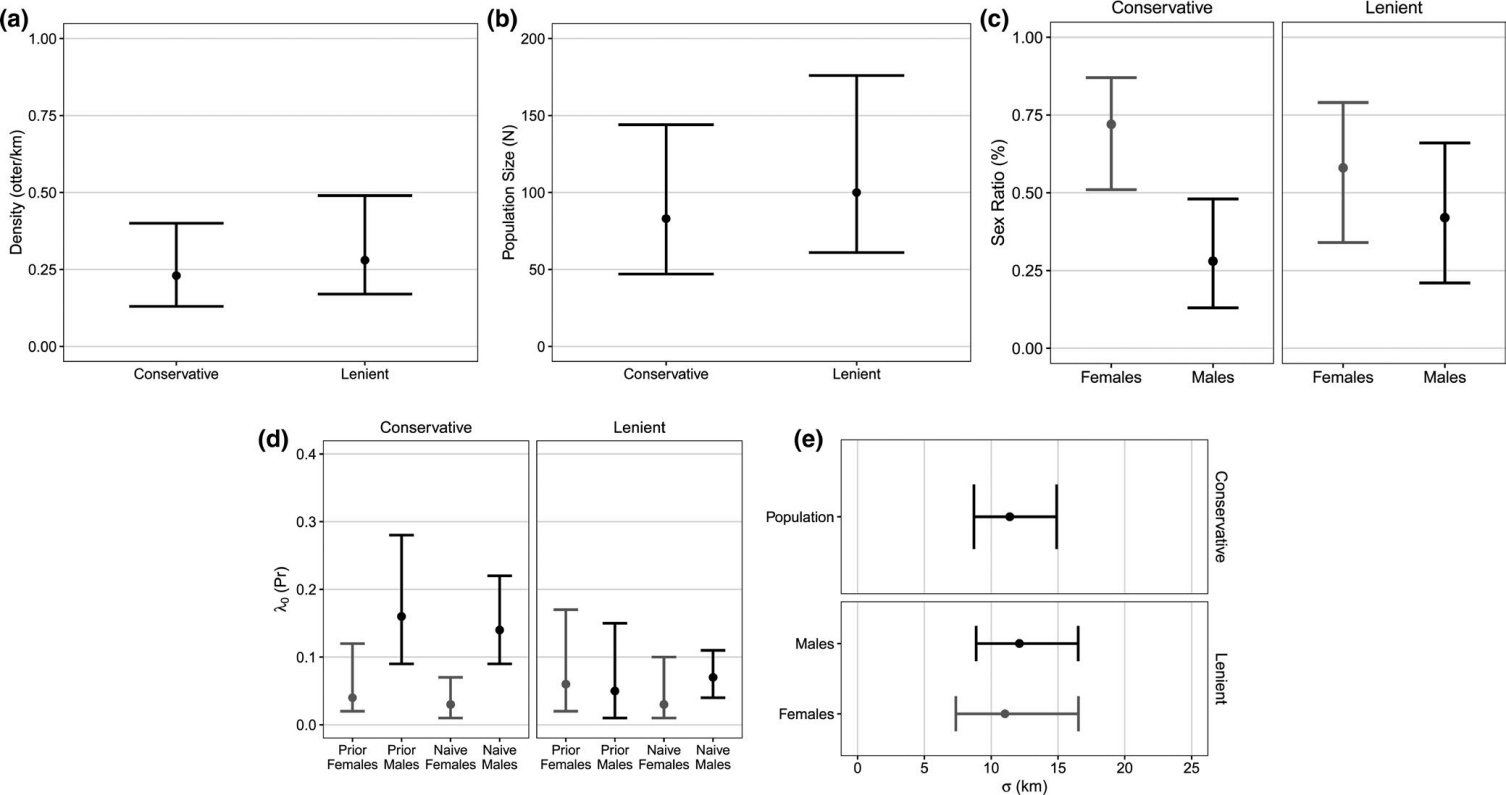
Parameter point estimates (**•**) and 95% confidence intervals from model‐averaging of competing (≤2 ΔAIC*_c_*) spatial capture–recapture models that were fitted to conservative and lenient river otter detection histories. The two detection histories were constructed based on genotype matching rules that reflected uncertainty about individual identification due to genotyping error. We estimated (a) population density, (b) population size, (c) population sex ratio, (d) the baseline detection rate, *λ*_0_, and (e) the spatial scale of detection, *σ*. Estimates of *λ*_0_ correspond to sex variation in a latrine‐specific behavioral response for otters that were detected at a latrine during prior survey occasions (Prior) or were not previously detected at a latrine (Naive). Corresponding numerical parameter estimates are provided in Appendix S2: Table B3

We model‐averaged competing models to produce final parameter estimates. Estimates of *σ* were similar between the two detection histories (11.39 vs. 11.03–12.11 km), which corresponded to an optimal buffer extent of ~50 km from latrines (Appendix S2: Figure B1), resulting in a 359‐km dendritic state space. The lenient detection history had 50%–69% lower estimates of male *λ*_0_ and 21.7% larger estimate of density compared with the conservative detection history but estimates of female *λ*_0_ were similar (Figure 4; Appendix S2: Table B3). The conservative estimate of population density was 0.23 otter/km (95% CI: 0.13–0.40), or 1 otter/4.35 km (95% CI: 2.50–7.69), whereas the lenient estimate was 0.28 otter/km (95% CI: 0.17–0.49), or 1 otter/3.57 km (95% CI: 2.04–5.88). The conservative sex ratio estimate was strongly female‐biased (0.72 F:0.28 M) but the lenient sex ratio estimate had a larger male component (0.58 F:0.42 M). The conservative population size estimate was 83 total otters (95% CI: 47–144), whereas the lenient population size estimate was 104 total otters (95% CI: 61–176). These estimates corresponded to conservative and lenient average annual exponential population growth rates during 2010–2018 of 1.12/year (95% CI: 1.05–1.20) and 1.15/year (95% CI: 1.08–1.23), respectively.

## 4 DISCUSSION

Although our estimates of spatially explicit river otter density (0.23–0.28 otter/km) for the URG population were within the range of reported densities for the species, they were toward the lower bound. River otter densities tend to vary between inland and coastal systems, with densities of 0.07–0.51 and 0.28–0.93 otter/km, respectively (Brzeski et al., 2013; Larivière & Walton, 1998; Melquist & Hornocker, 1983). This discrepancy between systems is primarily the result of coastal bays generally providing higher quantities of suitable habitats and food resources than inland rivers and streams (Blundell et al., 2001; Kruuk, 1995). Nevertheless, comparisons among our density estimates and those reported in the literature must be interpreted with caution. Previous river otter densities were derived either from track counts and other indices or from population sizes that were estimated using traditional non‐spatial capture–recapture models. The former are rarely reflective of true otter population size or density (Gallant et al., 2007; Quaglietta et al., 2015; Rivera et al., 2019), and densities from the latter are often positively biased, largely because the area to which population size estimates apply is unknown and must be approximated using ad hoc methods (Obbard et al., 2010; Sutherland et al., 2016). In contrast, SCR models explicitly define the geographical area to which estimates apply and can produce unbiased estimates of population density.

To our knowledge, this is the first study to estimate population size and density for any otter species using SCR models with a non‐Euclidean distance model specification that reflected typical otter movements within a branched dendritic network of waterways. We are aware of only one previous but unpublished study that estimated otter density with SCR models (Forman, 2015); however, that study used the default Euclidean distance model that assumes home ranges are approximately circular and applied a convex hull state space that included all terrestrial lands in the intervening areas among waterways. Although otters can traverse terrestrial habitats that are interspersed between aquatic habitats, they do so infrequently relative to movements along waterways (Carranza et al., 2012; Sauer et al., 1999). Even in hydric landscapes where multiple waterbodies are spatially proximal, otter movements predominantly occur along the dendritic network and result in approximately linear home ranges (Quaglietta et al., 2015; Sauer et al., 1999). However, using a convex hull state space and a Euclidean distance model results in activity center locations being estimated in terrestrial areas with similar probability as being estimated within the dendritic network, which inaccurately describes typical otter space use and can substantially bias population size and density estimates (Efford, 2019; Sutherland et al., 2015). Furthermore, if the array of detectors (latrines) is preferentially located along the dendritic network such that the sampling distribution is aligned with the primary directions of animal movement, as is common in otter studies, additional estimate bias will exist if a detection function transformation is not used (Efford, 2019; Murphy et al., 2016). In contrast, the network distance approach combined with a dendritic state space overcomes these issues for populations in dendritic networks and reflects the ecological realities of animal movement within them, thereby improving parameter estimates (Efford, 2019; Leuenberger et al., 2019; Warbington & Boyce, 2020).

Nevertheless, our population estimates may not be completely free from bias, given genotyping error was present. Genotyping error may be more prevalent in scat from otters and other piscivorous mammals, because the high lipid and low fiber content of consumed meats reduces intestinal cell slough rates (Murphy et al., 2003), and the digestive by‐products of aquatic fauna can interfere with the chemistry of DNA extraction protocols (Aristizábal Duque et al., 2018; Lerone et al., 2014). Brzeski et al. (2013) encountered a 28.6% allelic dropout rate when genotyping fecal DNA from river otter scats in California, USA, similar to our 29% allelic dropout rate. However, this was an averaged rate/locus/PCR and we also conducted four to six PCR replicates per sample before determining a consensus genotype; thus, genotyping error rates for our final genotypes should average <0.007 per locus.

Approaches for accommodating genotyping error in SCR models using the spatial partial‐identity and random thinning classes of models were recently developed (Augustine et al., 2019, 2020; Jiménez et al., 2021), but those methods have not yet been extended to detection data from populations with predominantly non‐circular home range orientations and non‐Euclidean movement (Warbington & Boyce, 2020). Therefore, we chose to produce parameter estimates from two sets of detection histories that reflected conservative and lenient genotype matching rules. We suspect that the population density, size, and therefore growth rate estimates based on the conservative dataset are more reliable, because genotyping error typically inflates the number of unique individuals and leads to overestimates of population size (Augustine et al., 2020; Knapp et al., 2009; Wright et al., 2009). We also found no or fewer violations of Hardy–Weinberg and linkage equilibrium with the conservative dataset.

Unfortunately, despite using sample collection, storage, and laboratory methods that were optimized for otters, we still incurred a low genotyping success rate (14%) that was also similar to other otter fecal DNA studies ($\bar{x}$ = 26%, range: 8%–60%; Mowry et al., 2011; Guertin et al., 2012; Brzeski et al., 2013). Although the effect of genotyping failure on SCR density estimates is functionally similar to randomized subsampling, which SCR models are robust to (Murphy et al., 2016), this still results in the loss of spatial recaptures, which degrades parameter estimate precision while also reducing the efficiency of non‐invasive genetic sampling (Augustine et al., 2019; Murphy et al., 2018). As noted for both scat and hair samples collected from other carnivores in the southwestern United States (Gould et al., 2018; Naidu et al., 2011), we suspect that high ultraviolet radiation in the region caused rapid scat decomposition and DNA degradation (Pilliod et al., 2014; Strickler et al., 2015). Survey occasions <7 days in duration may be necessary to combat conditions present in the Southwest; although scat accumulation rates at latrines tend to be slow for otters (Gallant et al., 2007; Rivera et al., 2019), so shorter occasion durations may result in fewer samples collected (Lonsinger et al., 2015). Additionally, recently developed alternative fecal DNA sampling methods, such as swabbing a scat with a cotton swab rinsed in DNA lysis buffer, may improve genotyping success rates for otters (Klütsch & Thomas, 2018); however, the swabbing method had inferior genotyping success rates for carnivore fecal samples collected in arid environments that were similar to our study area (Miles et al., 2015).

The strength of individual‐level site fidelity relative to home range size may also influence SCR model parameter estimates. When site fidelity is strong and home range sizes are large, non‐independence of detections can occur that results in spatial clustering of detections around the centroid of the *portion* of a home range used during survey occasions rather than the entire seasonal or annual home range (Royle et al., 2015). Otters are assumed to have high site fidelity to multiple latrines within a home range, but that has been primarily founded on camera‐trapping or scat sampling absent individual identification and telemetry data from often small sample sizes of radio‐marked individuals (Gorman et al., 2006; Rivera et al., 2019; Stevens & Serfass, 2008). To our knowledge, this study is the first to attempt to estimate individual‐level, sex‐specific latrine site fidelity for otters from structured capture–recapture detection data. Consequently, no estimates from other populations are available to which we can make informed comparisons, but our modification of the composite population‐level SSFI that was developed for capture–recapture data (Tschopp et al., 2018) could be used by other researchers in future studies (or applied to data from previously published studies) to obtain individual‐level latrine site fidelity for comparisons. We found that, despite differential latrine use between the sexes, latrine site fidelity was low for both sexes based on both the conservative and lenient detection data (SSFI <0.15), and perhaps even nominal based on the lenient detection data (95% CIs included zero). Therefore, it is unlikely that latrine site fidelity was strong enough to cause non‐independence or clustering of detections at a level substantial enough to influence SCR model parameter estimates, though we caution that the poor genotyping success rate that we encountered likely resulted in lost detections, which could negatively bias the SSFI calculation. Nevertheless, based on both simulation and empirical data, Royle et al. (2015) found that SCR density estimates were robust to spatial clustering of detections caused by non‐independence, but that under‐coverage of confidence intervals and biased estimates of the scale parameter, *σ*, can occur. However, those findings applied to the typical SCR Euclidean distance movement model while assuming stationarity of activity centers and it remains unclear what, if any, translatability exists to SCR models that accommodate non‐Euclidean movement.

Optimistically assuming all 33 founder otters survived the founder event and that exponential growth was possible, the reintroduced URG population has exhibited moderate average annual population growth. However, our optimistic growth rates were ≥40% lower than most rates estimated for other reintroduced river otter populations (e.g., Barding & Lacki, 2014; Breitenmoser et al., 2001; Ellington et al., 2018). Although a feasibility study indicated that the URG dendritic network was the most suitable for otters relative to other river systems in New Mexico (NMDGF, 2006), most reintroduced river otter populations were established using founder groups that were substantially larger than in the URG (range: 123–845 founders; Mowry et al., 2015; Raesly, 2001; Roberts et al., 2020). Initially, the small founder group presumably had fewer breeding opportunities compared with larger founder groups, which likely impeded population growth during the initial establishment phase. An unfortunate consequence of the small founder group is a bottlenecking founder effect that reduced genetic diversity and genetic effective sizes, the latter of which were below the minimum that may be necessary for long‐term population viability (*N*_E_ > 50; Frankham et al., 2014). The compounding effect of lasting isolation and subsequent genetic drift also has led to rapid divergence from the source population (*F*_ST_ accumulation = 0.06/generation, assuming $\bar{x}$ generation time = 6.4 years [Boyle, 2006; Mowry et al., 2015]). Thus, considering the small population size and isolation, genetic restoration via additional translocations of otters from other populations may be required to prevent further genetic degradation in this small population; although, the estimated female‐biased sex ratio suggests that population growth may continue, which could mitigate additional genetic diversity loss (Groombridge et al., 2012; Murphy et al., 2015, 2016).

Accurately assessing the demographic and genetic statuses of reintroduced populations can be challenging, particularly if populations were established using small founder groups (Ewen & Armstrong, 2007; Nichols & Armstrong, 2012). For such assessments, the influence that habitat or landscape structure may have on populations requires careful consideration in study design development and appropriate accommodations in analytical methods to produce reliable estimates for parameters of interest. With species‐specific sampling modifications, the SCR network distance approach should be widely applicable to multiple populations of mustelid species that inhabit aquatic dendritic networks. For example, the critically endangered European mink (*Mustela lutreola*) and endangered hairy‐nosed otter (*Lutra sumatrana*) inhabit riverine dendritic networks, but population density and size estimates that are critical to their conservation have not yet been produced. We importantly note that if the branches of dendritic networks are numerous and spatially proximal, such that the distances between branches are conducive to ‘shortcut’ animal movements across the intervening terrestrial areas instead of primarily along the dendritic network, then the SCR ecological distance model likely would be more appropriate than the network distance function (Royle et al., 2013; Sutherland et al., 2015). That approach was successfully applied to estimate population size and density of a similar mustelid, the American mink (*Neovison vison*), in the northeastern United States, where mink space use was associated with riverine dendritic networks that had multiple spatially proximal branches, but space use was not confined solely to within the network (Fuller et al., 2016; Sutherland et al., 2018). However, estimate precision of the ecological distance model degrades as the strength of association between animal movement and environmental structure increases (Sutherland et al., 2015), rendering the network distance approach optimal when animal movement is confined primarily to within dendritic networks.

## CONFLICT OF INTEREST

Sean M. Murphy was employed by the primary funding entity, New Mexico Department of Game & Fish. All other authors have no conflicts of interest to declare.

## AUTHOR CONTRIBUTION

**Sean M. Murphy:** Conceptualization (equal); Data curation (equal); Formal analysis (lead); Investigation (equal); Methodology (equal); Resources (equal); Validation (equal); Visualization (lead); Writing‐original draft (equal); Writing‐review & editing (equal). **Jennifer R. Adams:** Formal analysis (equal); Methodology (equal); Resources (equal); Validation (equal); Writing‐review & editing (equal). **Lisette P. Waits:** Formal analysis (equal); Methodology (equal); Resources (equal); Validation (equal); Writing‐review & editing (equal). **John J. Cox:** Conceptualization (equal); Data curation (equal); Formal analysis (equal); Funding acquisition (lead); Investigation (equal); Methodology (equal); Project administration (lead); Resources (lead); Supervision (lead); Validation (equal); Visualization (equal); Writing‐original draft (equal); Writing‐review & editing (equal).

### OPEN RESEARCH BADGES

This article has earned an Open Data Badge for making publicly available the digitally‐shareable data necessary to reproduce the reported results. The data is available at https://doi.org/10.5061/dryad.kkwh70s51

## Supporting information

Appendix S1

Appendix S2

## Data Availability

All data used in this study are available in the Dryad repository at: https://doi.org/10.5061/dryad.kkwh70s51.
