# Supplementary material for: Evaluating otter reintroduction outcomes using genetic spatial capture–recapture modified for dendritic networks
Source: Ecol Evol. 2021 Oct 7;11(21):15047–61. doi: 10.1002/ece3.8187 (PMC8571598; doi:10.1002/ece3.8187)
Supplement: Supplementary file 1 — Appendix S1 [file ECE3-11-15047-s001.docx]

**Appendix S1: Estimates of probability of identity for siblings (*PI*sibs) and genotyping error rates for river otter genotypes from fecal samples collected in New Mexico, USA (2018).**

Correspondence:

Sean M. Murphy, Ph.D.

Department of Forestry and Natural Resources

University of Kentucky

214 T.P. Cooper Building

Lexington, KY 40546, USA

Tel: +1 276 393 1360. Email: [smmurp2@uky.edu](mailto:smmurp2@uky.edu)

John J. Cox, Ph.D.

Department of Forestry and Natural Resources

University of Kentucky

102 T.P. Cooper Building

Lexington, KY 40546, USA

Tel: +1 859 257 9501. Email: [jjcox@uky.edu](mailto:jjcox@uky.edu)

**Table A1.** Per locus probability of identity for siblings (*PI*sibs) calculated for tissue samples from the source population (WA) and fecal samples from the reintroduced population (URG).

| Population | Microsatellite Loci | | | | | | | | | |
| --- | --- | --- | --- | --- | --- | --- | --- | --- | --- | --- |
|  | Lut453 | RIO01 | RIO02 | RIO04 | RIO06 | RIO07 | RIO08 | RIO12 | RIO13 | RIO16 |
| WA Source | 0.45 | 0.41 | 0.51 | 0.40 | 0.49 | 0.43 | 0.52 | 0.50 | 0.39 | 0.40 |
| URG Conservative | 0.63 | 0.49 | 0.58 | 0.63 | 0.66 | 0.53 | 0.68 | 0.48 | 0.39 | 0.44 |
| URG Lenient | 0.64 | 0.51 | 0.61 | 0.63 | 0.71 | 0.54 | 0.66 | 0.49 | 0.39 | 0.44 |

**Table A2.** Probability of identity for siblings (*PI*sibs) calculated at 6, 7, 8, and 9 loci for tissue samples from the source population (WA) and fecal samples from the reintroduced population (URG).

| Population | Number of Loci | | | | | | | |
| --- | --- | --- | --- | --- | --- | --- | --- | --- |
|  | 6 Best | 6 Worst | 7 Best | 7 Worst | 8 Best | 8 Worst | 9 Best | 9 Worst |
| WA Source | 0.005 | 0.013 | 0.002 | 0.005 | 0.001 | 0.002 |  |  |
| URG Conservative | 0.012 | 0.055 | 0.008 | 0.027 | 0.005 | 0.013 | 0.003 | 0.006 |
| URG Lenient | 0.014 | 0.062 | 0.009 | 0.032 | 0.006 | 0.016 | 0.004 | 0.007 |

**Table A3.** Per locus genotyping error rates for fecal samples from the reintroduced population (URG).

| Error | Microsatellite Loci | | | | | | | | | |
| --- | --- | --- | --- | --- | --- | --- | --- | --- | --- | --- |
|  | Lut453 | RIO01 | RIO02 | RIO04 | RIO06 | RIO07 | RIO08 | RIO12 | RIO13 | RIO16 |
| Allelic Dropout | 0.27 | 0.25 | 0.32 | 0.44 | 0.38 | 0.30 | 0.19 | 0.28 | 0.20 | 0.28 |
| False Alleles | 0.04 | 0.02 | 0.10 | 0.04 | 0.00 | 0.13 | 0.03 | 0.02 | 0.05 | 0.02 |
