# Supplementary material for: Evaluating otter reintroduction outcomes using genetic spatial capture–recapture modified for dendritic networks
Source: Ecol Evol. 2021 Oct 7;11(21):15047–61. doi: 10.1002/ece3.8187 (PMC8571598; doi:10.1002/ece3.8187)
Supplement: Supplementary file 2 — Appendix S2 [file ECE3-11-15047-s002.docx]

**Appendix S2: Spatial capture-recapture (SCR) modeling results from analysis of fecal-based river otter detections in New Mexico, USA (2018).**

Correspondence:

Sean M. Murphy, Ph.D.

Department of Forestry and Natural Resources

University of Kentucky

214 T.P. Cooper Building

Lexington, KY 40546, USA

Tel: +1 276 393 1360. Email: [smmurp2@uky.edu](mailto:smmurp2@uky.edu)

John J. Cox, Ph.D.

Department of Forestry and Natural Resources

University of Kentucky

102 T.P. Cooper Building

Lexington, KY 40546, USA

Tel: +1 859 257 9501. Email: [jjcox@uky.edu](mailto:jjcox@uky.edu)

**Table B1.** Spatial capture-recapture model selection from analysis of conservative detection histories for the reintroduced river otter population in the Upper Rio Grande dendritic network (2018). Primary model parameters were population density (*D*), baseline detection rate (λ_0_), and the spatial scale of detection (σ). Models were fit in which *D* followed a homogenous Poisson point process (1) or spatially varied as a log-linear function of latitude (Lat). Models also considered a latrine-specific behavioral response (*bk*) that was either shared between sexes or was sex-specific (Sex), via both additive (+) and interaction (×) effects, and considered σ that was either sex-specific (Sex) or shared between sexes (1).

| Model | *K*^a^ | AIC^b^ | AIC*_c_*^c^ | ΔAIC*_c_*^d^ | logLik^e^ | *w*^f^ |
| --- | --- | --- | --- | --- | --- | --- |
| *D*~1 λ_0_~Sex σ~1 | 5 | 527.03 | 529.53 | 0.00 | –258.17 | 0.31 |
| *D*~1 λ_0_~*bk* + Sex σ~1 | 6 | 526.92 | 530.56 | 1.04 | –257.14 | 0.18 |
| *D*~Lat λ_0_~Sex σ~1 | 6 | 527.48 | 531.13 | 1.60 | –257.48 | 0.14 |
| *D*~1 λ_0_~*bk* × Sex σ~1 | 7 | 527.57 | 531.23 | 1.70 | –257.79 | 0.10 |
| *D*~1 λ_0_~ Sex σ~Sex | 6 | 528.60 | 532.25 | 2.72 | –258.30 | 0.08 |
| *D*~Lat λ_0_~*bk* + Sex σ~1 | 7 | 527.28 | 532.37 | 2.84 | –256.64 | 0.07 |
| *D*~1 λ_0_~*bk* + Sex σ~Sex | 7 | 528.55 | 533.65 | 4.11 | –257.28 | 0.05 |
| *D*~Lat λ_0_~*bk* × Sex σ~1 | 8 | 527.07 | 533.92 | 4.39 | –255.53 | 0.03 |
| *D*~Lat λ_0_~Sex σ~Sex | 7 | 529.14 | 534.23 | 4.70 | –257.57 | 0.02 |
| *D*~1 λ_0_~*bk* × Sex σ~Sex | 8 | 528.73 | 535.59 | 6.05 | –256.37 | 0.01 |
| *D*~Lat λ_0_~*bk* + Sex σ~Sex | 8 | 529.01 | 535.87 | 6.33 | –256.50 | 0.00 |
| *D*~Lat λ_0_~*bk* × Sex σ~Sex | 9 | 529.07 | 538.07 | 8.53 | –255.53 | 0.00 |
| *D*~1 λ_0_~1 σ~Sex | 5 | 536.12 | 538.62 | 9.09 | –263.06 | 0.00 |
| *D*~1 λ_0_~*bk* σ~Sex | 6 | 536.08 | 539.73 | 10.20 | –262.04 | 0.00 |
| *D*~Lat λ_0_~1 σ~Sex | 6 | 536.79 | 540.45 | 10.91 | –262.40 | 0.00 |
| *D*~Lat λ_0_~*bk* σ~Sex | 7 | 536.65 | 541.74 | 12.21 | –261.33 | 0.00 |
| *D*~1 λ_0_~*bk* σ~1 | 5 | 540.61 | 543.11 | 13.58 | –265.30 | 0.00 |
| *D*~1 λ_0_~1 σ~1 | 4 | 541.58 | 543.18 | 13.64 | –266.79 | 0.00 |
| *D*~Lat λ_0_~1 σ~1 | 5 | 541.88 | 544.38 | 14.85 | –265.94 | 0.00 |
| *D*~Lat λ_0_~*bk* σ~1 | 6 | 540.77 | 544.42 | 14.89 | –264.38 | 0.00 |

^a^ Number of model parameters.

^b^ Akaike’s Information Criterion.

^c^ AIC corrected for small sample size.

^d^ Difference between AIC*_c_* of model and AIC*_c_* of top ranked model.

^e^ log-likelihood.

^f^ Model weight.

**Table B2.** Spatial capture-recapture model selection from analysis of lenient detection histories for the reintroduced river otter population in the Upper Rio Grande dendritic network (2018). Primary model parameters were population density (*D*), baseline detection rate (λ_0_), and the spatial scale of detection (σ). Models were fit in which *D* followed a homogenous Poisson point process (1) or spatially varied as a log-linear function of latitude (Lat). Models also considered a latrine-specific behavioral response (*bk*) that was either shared between sexes or was sex-specific (Sex), via both additive (+) and interaction (×) effects, and considered σ that was either sex-specific (Sex) or shared between sexes (1).

| Model | *K*^a^ | AIC^b^ | AIC*_c_*^c^ | ΔAIC*_c_*^d^ | logLik^e^ | *w*^f^ |
| --- | --- | --- | --- | --- | --- | --- |
| *D*~1 λ_0_~Sex σ~1 | 5 | 545.16 | 549.02 | 0.00 | –265.58 | 0.19 |
| *D*~1 λ_0_~*bk* × Sex σ~1 | 7 | 547.56 | 549.49 | 0.47 | –268.78 | 0.15 |
| *D*~1 λ_0_~1 σ~Sex | 5 | 548.18 | 550.11 | 1.09 | –269.09 | 0.11 |
| *D*~1 λ_0_~1 σ~1 | 4 | 549.47 | 550.72 | 1.70 | –270.73 | 0.08 |
| *D*~Lat λ_0_~*bk* × Sex σ~1 | 8 | 546.01 | 551.15 | 2.13 | –265.00 | 0.07 |
| *D*~Lat λ_0_~ Sex σ~1 | 6 | 548.41 | 551.21 | 2.19 | –268.20 | 0.06 |
| *D*~1 λ_0_~ Sex σ~Sex | 6 | 548.79 | 551.59 | 2.57 | –268.39 | 0.05 |
| *D*~Lat λ_0_~1 σ~Sex | 6 | 549.12 | 551.92 | 2.91 | –268.56 | 0.04 |
| *D*~Lat λ_0_~1 σ~1 | 5 | 550.25 | 552.18 | 3.16 | –270.12 | 0.04 |
| *D*~1 λ_0_~*bk* × Sex σ~Sex | 8 | 547.14 | 552.28 | 3.26 | –265.57 | 0.04 |
| *D*~1 λ_0_~*bk* + Sex σ~1 | 6 | 549.54 | 552.34 | 3.32 | –268.77 | 0.04 |
| *D*~1 λ_0_~*bk* σ~Sex | 6 | 550.10 | 552.90 | 3.88 | –269.05 | 0.03 |
| *D*~1 λ_0_~*bk* σ~1 | 5 | 551.44 | 553.37 | 4.35 | –270.72 | 0.02 |
| *D*~Lat λ_0_~Sex σ~Sex | 7 | 549.72 | 553.58 | 4.57 | –267.86 | 0.02 |
| *D*~Lat λ_0_~*bk* + Sex σ~1 | 7 | 550.40 | 554.26 | 5.24 | –268.20 | 0.01 |
| *D*~1 λ_0_~*bk* + Sex σ~Sex | 7 | 550.75 | 554.62 | 5.60 | –268.38 | 0.01 |
| *D*~Lat λ_0_~*bk* × Sex σ~Sex | 9 | 548.00 | 554.67 | 5.65 | –265.00 | 0.01 |
| *D*~Lat λ_0_~*bk* σ~Sex | 7 | 551.07 | 554.93 | 5.91 | –268.53 | 0.01 |
| *D*~Lat λ_0_~*bk* σ~1 | 6 | 552.23 | 555.03 | 6.01 | –270.11 | 0.01 |
| *D*~Lat λ_0_~*bk* + Sex σ~Sex | 8 | 551.70 | 556.84 | 7.83 | –267.85 | 0.00 |

^a^ Number of model parameters.

^b^ Akaike’s Information Criterion.

^c^ AIC corrected for small sample size.

^d^ Difference between AIC*_c_* of model and AIC*_c_* of top ranked model.

^e^ log-likelihood.

^f^ Model weight.

**Table B3.** Parameter estimates and corresponding standard errors (SE) and 95% confidence intervals (CI) from model-averaging of competing (≤2 ΔAIC*_c_*) spatial capture-recapture models that were fitted to conservative and lenient river otter detection histories. The two detection histories were constructed based on genotype matching rules that reflected uncertainty about individual identification due to genotyping error. The baseline detection rate (λ_0_), spatial scale of detection (σ), population density, population size (abundance), and sex ratio were estimated. Estimates of λ_0_ correspond to sex variation in a latrine-specific behavioral response for otters that were detected at a latrine during prior survey occasions (Prior) or were not previously detected at a latrine (Naive).

| Parameter | Estimate | SE | 95% CI |
| --- | --- | --- | --- |
| Conservative |  |  |  |
| λ_0_ Female Naïve | 0.03 | 0.01 | 0.01 – 0.07 |
| λ_0_ Female Prior | 0.04 | 0.02 | 0.02 – 0.12 |
| λ_0_ Male Naïve | 0.14 | 0.03 | 0.09 – 0.22 |
| λ_0_ Male Prior | 0.16 | 0.05 | 0.09 – 0.28 |
| σ (km) | 11.39 | 1.57 | 8.71 – 14.90 |
| Density (otter/km) | 0.23 | 0.07 | 0.13 – 0.40 |
| Abundance (otters) | 83 | 25 | 47 – 144 |
| Female Ratio | 0.72 | 0.09 | 0.51 – 0.87 |
| Male Ratio | 0.28 | 0.09 | 0.13 – 0.49 |
| Lenient |  |  |  |
| λ_0_ Female Naïve | 0.03 | 0.02 | 0.01 – 0.10 |
| λ_0_ Female Prior | 0.06 | 0.03 | 0.02 – 0.17 |
| λ_0_ Male Naïve | 0.07 | 0.02 | 0.04 – 0.11 |
| λ_0_ Male Prior | 0.05 | 0.03 | 0.01 – 0.15 |
| σ Female (km) | 11.03 | 2.30 | 7.36 – 16.53 |
| σ Male (km) | 12.11 | 1.93 | 8.88 – 16.51 |
| Density (otter/km) | 0.29 | 0.08 | 0.17 – 0.49 |
| Abundance (otters) | 104 | 29 | 61 – 176 |
| Female Ratio | 0.58 | 0.12 | 0.34 – 0.79 |
| Male Ratio | 0.42 | 0.12 | 0.21 – 0.66 |

**Figure B1.** Plots of hazard half-normal detection functions from spatial capture-recapture models that were fit to conservative and lenient river otter detection histories. The probability of detecting a river otter at a surveyed latrine was effectively zero if the otter’s activity center was located >50 km away from the latrine.
